# Influence of Various Model Compounds on the Rheological Properties of Zein-Based Gels

**DOI:** 10.3390/molecules25143174

**Published:** 2020-07-11

**Authors:** Agnese Gagliardi, Silvia Voci, Donatella Paolino, Massimo Fresta, Donato Cosco

**Affiliations:** 1 Department of Experimental and Clinical Medicine, University “Magna Græcia” of Catanzaro, Campus Universitario “S. Venuta”, I-88100 Catanzaro, Italy; gagliardi@unicz.it (A.G.); paolino@unicz.it (D.P.); 2 Department of Health Sciences, University “Magna Græcia” of Catanzaro, Campus Universitario “SVenuta”, I-88100 Catanzaro, Italy; silvia.voci@studenti.unicz.it (S.V.); fresta@unicz.it (M.F.)

**Keywords:** controlled release, gels, polymeric matrix, probes, rheology, zein

## Abstract

The controlled release of a compound entrapped in a biocompatible formulation is a sought-after goal in modern pharmaceutical technology. Zein is a hydrophobic protein which has several advantageous properties that make it suitable for use as a biocompatible and degradable material under physiological conditions. It is, therefore, proposed for different biomedical and pharmaceutical applications. In particular, due to its gelling properties, it can be used to form a polymeric network able to preserve biomolecules from harsh environments. The current study was designed to investigate the influence of different probes on the rheological properties of gels made up of zein, in order to characterize the systems as a function of the polymer concentration. Four model compounds characterized by different physico-chemical properties were entrapped in zein gels, and different behaviors (viscoelastic or pronounced solid-like characteristics) of the systems were observed. Zein-based gels showed various release profiles of the encapsulated compounds, suggesting that there are different interaction rates between the probes and the polymeric matrix.

## 1. Introduction

The impact of natural polymers on the pharmaceutical and biomedical fields is exponentially increasing. In this context, zein, a prolamin-rich protein contained in the endosperm of corn kernels, is one of the most promising and versatile biopolymers due to its soft and versatile nature, significant availability, and because it can be obtained from renewable and inexpensive sources [1]. In addition, it is a biomaterial that has been generally recognized as safe (GRAS) by the U.S. Food and Drug Administration (FDA) because it displays good standards of biocompatibility and biodegradability while having only a low degree of toxicity [2,3]. Zein is a mixture of proteins and, according to its solubility and sequence homology, it currently has four classifications: α-zein (19 and 22 kDa, the highest content of zein mass); β-zein (14 kDa); γ-zein (16 and 27 kDa), and δ-zein (10 kDa) [4,5,6,7]. It contains a rich amount of nonpolar aminoacid residues, which are responsible for its highly hydrophobic properties and characteristics of solubility.

The hydrophobicity of protein is 50 times greater than albumin or fibrinogen [8]. Indeed, it is fairly abundant in glutamic acid, leucine, proline, and alanine, together with a little arginine and histidine and some polar aminoacid residues such as glutamine. However, it is lacking in tryptophan and lysine as compared to other proteins [6]. This composition of aminoacids promotes a unique solubility of zein in aqueous alcohol, alkaline solutions, high concentrations of urea or anionic surfactants [9]. The composition and solubility of zein are prohibitive for its application in food products for human consumption, due to the poor nutritional quality of the protein [2]. However, the unique properties of this protein have great potential as a starting biomaterial for the development of various structures, including micro/nanoparticles, films, gels, and other formulations able to achieve a sustained release of several drugs as well as improving their therapeutic efficiency and patient compliance [9,10,11,12,13,14,15,16].

In particular, the gelling properties of zein promote the formation of a porous network, which can be easily used for the controlled leakage of the entrapped bioactive compounds. Several studies have described the rheological behavior of specific fractions of zein such as α- and γ-zein dispersions, obtained after the purification of the protein [17,18,19], and different materials such as glycerol, β-carotene, acrylic acid, propylene glycol alginate, tannic acid, pea protein, citric acid and acetic anhydride were added to modulate the gelling features of zein [20,21,22,23,24,25]. However, our research team recently used commercial zein, at a considerably lower cost than that of the purified form, to prepare gels (up to 20% *w*/*v* of protein) without any chemical refinement or the addition of gelling agents; for the first time, the characterization of zein gels was performed under static and dynamic conditions [26]. During the phases of characterization of a formulation, the effects of the compound(s) to be entrapped on the characteristics of the system need to be evaluated; for this reason, different molecules were added to the zein gels in order to investigate their impact on the features of these protein systems. In detail, disodium fluorescein and rhodamine B were used as a model of a hydrophilic compound, while methylene blue and bromophenol blue were chosen as amphiphilic and lipophilic compounds, respectively. The aforementioned molecules are fluorescent dyes routinely used to stain biological tissues [27] as well as to investigate drug interaction in the liver [28,29]. Among them, methylene blue represents a compound widely known in clinical practice [30]. Its cationic nature promotes significant photo-antimicrobial activity, particularly against Gram-positive bacteria such as Propionibacterium acnes [31,32]. It is commonly used for various applications such as the treatment of malaria and methemoglobinemia (Provayblue^®^), prevention of urogenital infections (Urolene Blue^®^) and ifosfamid-induced encephalopathy, and treatment of septic shock and priapism, as well as the sterilization of blood transfusions [33].

Based on these findings, the current study was designed to investigate the influence of the aforesaid probes characterized by different physico-chemical properties on the rheological properties of zein-based gels. Finally, the release profiles of the compounds from the protein network were evaluated and the potential interaction between the probe and zein gels was discussed.

## 2. Materials and Methods

### 2.1. Materials

Zein (CAS number 9010-66-62, EC number: 232-722-9), rhodamine B, disodium fluorescein, bromophenol blue, methylene blue, and ethanol were purchased from Sigma-Aldrich (Milan, Italy). All other materials and solvents used in this investigation were of analytical grade (Carlo Erba, Milan, Italy), while cellulose membrane Spectra/Por MWCO 50 kDa was obtained from Spectrum Laboratories Inc. (Eindhoven, The Netherlands).

### 2.2. Preparation of Zein Gels

Zein gels were prepared by the addition of various amounts of biopolymer (15%, 20% *w*/*v*) to a EtOH/H_2_O solution (35:65, %*v/v*) under constant, slow magnetic stirring at room temperature to favor the evaporation of the organic solvent and the formation of the gel [26].

Zein gels containing different model compounds (rhodamine B, disodium fluorescein, bromophenol blue, methylene blue) were obtained by adding different amounts of probes (0.5%, 1%, 2.5%, 5% *w*/*w* with respect to the protein) in water or ethanol during the sample preparation as a function of their chemical properties (Table 1).

### 2.3. Rheological Measurements of Zein Gels

The microrheological analysis of zein gels was performed through diffusive wave spectroscopy (DWS) with a microrheometer Rheolaser Master (Formulaction, I’Union, Toulouse, France) and expressed as solid-liquid balance (SLB) value, which provides information about the solid-like and liquid-like character of the sample as a function of time, as previously reported [26,34,35]. In detail, a value of 0 < SLB < 0.5 indicates a solid behavior, while a value of 0.5 < SLB < 1 means that the liquid behavior is predominant (Rheolaser Master™ user guide, Formulaction, France).

Dynamic rheological tests were carried out on zein-based gels containing different model compounds by means of a Kinexus rheometer (Malvern Instruments Ltd., Worcestershire, UK) using a cone-plate geometry. To assure suitable experimental conditions for the rheological measurements inside the linear viscoelasticity region (LVR), strain sweep tests ranging from 0.01 to 100% were performed at a frequency of 1 Hz (Figure S1). Frequency sweep measurements were then applied between 0.1 and 100 Hz by keeping the strain constant at 1% in order to obtain rheological parameters such as storage or elastic modulus (G′) and loss or viscous modulus (G″) of zein-based gels. Furthermore, the viscosity profiles of samples as a function of shear rate in the range of 0.1–100 s^−1^ were determined [36].

### 2.4. In Vitro Release of Model Compounds from Zein Gels

The leakage of probes from the zein-based gels was evaluated by the dialysis method using cellulose acetate tubes (Spectra/Por with molecular cutoff 50k by Spectrum Laboratories Inc., Eindhoven, The Netherlands) and a PBS solution (pH 7.4, 0.1 M) constantly stirred and warmed at 37 °C as the release fluid for the compounds. In detail, 1 mL of each final formulation of zein gel was placed in the dialysis bag (surface area of ~24 cm^2^) sealed at both ends with clips. The dialysis bag was attached vertically, fully stretched, then immersed in a beaker containing 200 mL of the release medium in order to operate under sink conditions for 160 h [37]. At predetermined time intervals, 1 mL of the release medium was withdrawn and replaced with fresh. The samples were then subjected to spectrophotometric analysis (Lambda 35; Perkin Elmer, Waltham, MA, USA) at λ_max_ 490 nm, 544 nm, 590 nm and 663 nm for disodium fluorescein, rhodamine B, bromophenol blue and methylene blue, respectively. No interference deriving from the empty zein formulation was observed.

### 2.5. Statistical Analysis

Statistical analysis of the various experiments was carried out through the ANOVA test.

## 3. Results and Discussion

### 3.1. Rheological Features of Zein Gels

**Figure 1:**
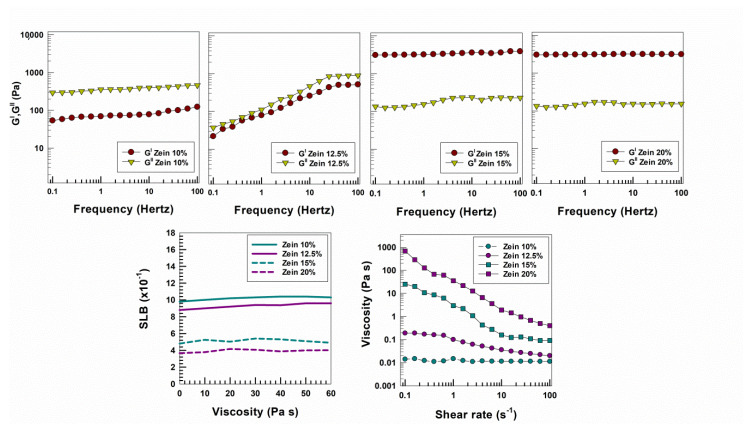
Evaluation of elastic (G′) and viscous (G″) moduli, solid liquid balance (SLB) and viscosity of zein formulations (made up of 10%, 12.5%, 15%, 20% *w*/*v* of protein) as a function of frequency, time and shear rate, respectively.

The rheological profiles of various dispersions of raw zein prepared as a function of the protein concentration were recently investigated and characterized by our research team in order to provide a panel of various formulations useful for food, biomedical and drug delivery applications [26]. As shown in Figure 1, the protein systems prepared by means of a zein concentration of 15% and 20% *w*/*v* showed values of the storage modulus that were significantly greater than those of the viscous modulus, demonstrating the prevalence of an elastic character and stable gels. These findings were also confirmed by a viscosity parameter that decreased when the shear rate increased, evidencing a typical pseudoplastic behavior; moreover, the passive micro-rheology analysis showed SLB values less than 0.5 for these dispersions, clearly demonstrating a solid-like profile [26]. These results are related to the physico-chemical features of the biopolymer which are able to rearrange as a function of the medium. Zhang and coworkers demonstrated that the rapid evaporation of ethanol and water causes the increase in the polarity of the environment; consequently, the zein molecules expose their hydrophilic residues towards the solvent, minimizing the surface tension [17]. For this reason, when a low concentration of zein was used (10% *w*/*v* and 12.5% *w*/*v*) the electrostatic forces prevailed as a consequence of the weaker polymeric intermolecular attraction, promoting the protein–water interaction. This trend influences the rheological features of the protein dispersions and makes the sample more liquid-like. Contrarily, when the zein concentration is increased (15% and 20% *w*/*v*) the hydrophobic interactions and Van der Waals forces could cause the formation of a rigid polymeric structure, as confirmed by the analyses of static and dynamic rheology (Figure 1).

**Figure 2:**
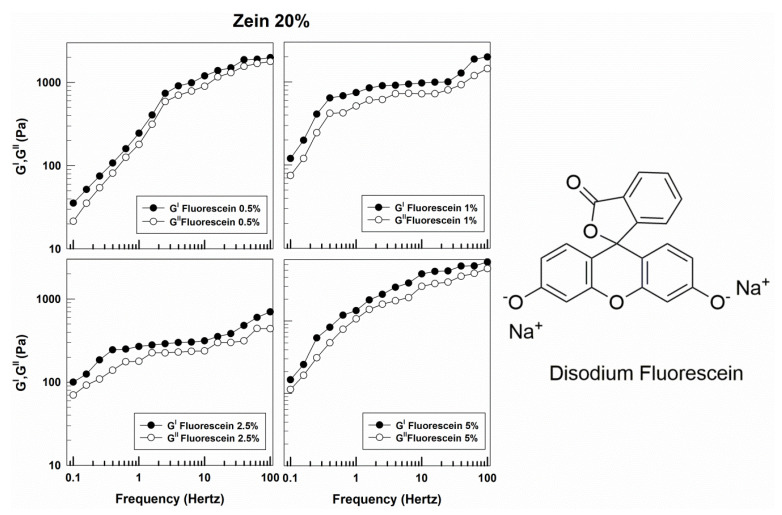
Evaluation of the elastic (G′) and viscous (G″) moduli of zein gels prepared using 20% *w*/*v* of protein and containing disodium fluorescein (0.5%, 1%, 2.5%, 5% *w*/*w*) as a function of the frequency.

**Figure 3:**
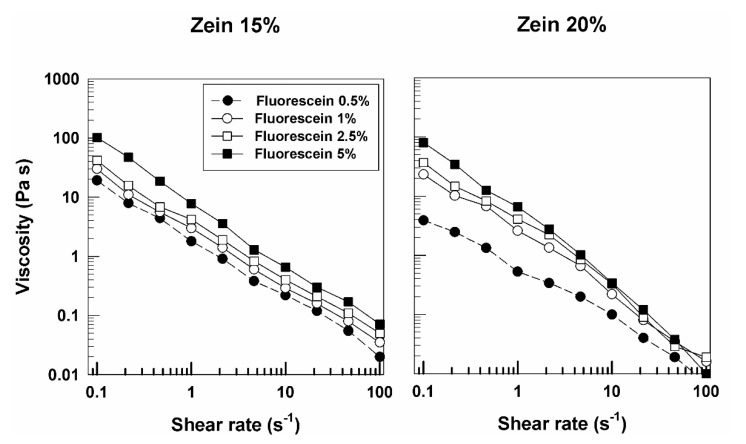
Evaluation of viscosity of zein samples containing disodium fluorescein as a function of the shear rate.

Considering the aforementioned features, the influence of the physico-chemical properties of a molecule on the rheological properties of zein gels should be investigated. Indeed, the viscoelastic characteristics of the pharmaceutical formulations used can dramatically influence both the residence time and the release rate of entrapped compounds at the application site [38]. As shown in Figure 2, various amounts of disodium fluorescein promoted a decrease in the gap between the elastic and the viscous moduli with respect to the empty formulation; the water-soluble nature of the salt probably promotes its localization within the hydrophilic residues of zein and the aqueous compartments of gel, favoring a decrease in the hydrophobic interactions of the polymeric matrix. This is why the samples showed comparable and frequency-dependent profiles of storage and loss moduli, suggesting a certain similarity to a viscoelastic material with a low degree of elasticity [39]. An analogous trend was obtained when 15% of zein was used to obtain the gel network, confirming the significant influence of the hydrophilic compound on the structural rearrangement of the gels (Figure S2). A corroboration of this hypothesis was provided by the evaluation of the sample viscosity (Figure 3); in fact, even though a pseudoplastic behavior was maintained by all the formulations, the decrease in the initial value of this parameter was noteworthy as compared to the empty system (Figure 3).

**Figure 4:**
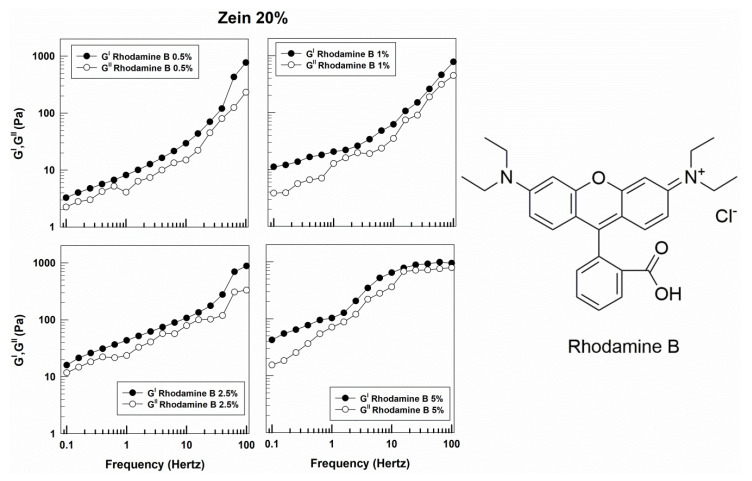
Evaluation of the elastic (G′) and viscous (G″) moduli of zein gels prepared using 20% *w*/*v* of protein and containing rhodamine B (0.5%, 1%, 2.5%, 5% *w*/*w*) as a function of the frequency.

**Figure 5:**
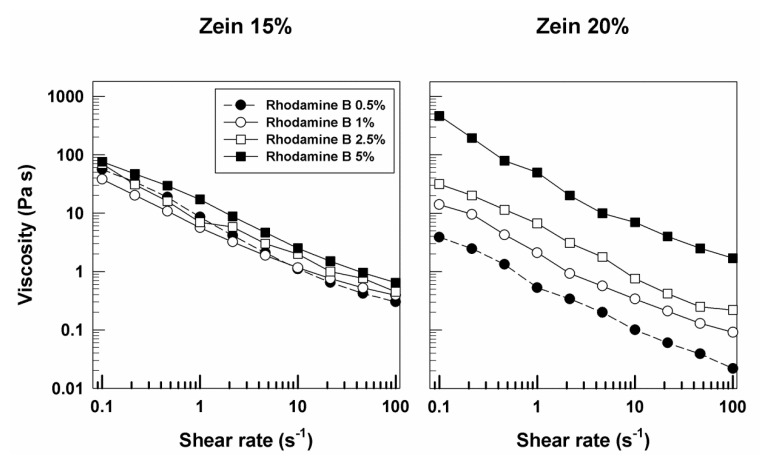
Evaluation of viscosity of zein samples containing rhodamine B as a function of the shear rate.

Similar rheological features were obtained when rhodamine B was used as model of hydrophilic compound instead of disodium fluorescein. The profiles of G′ and G″ were close, with a prevalence of the elastic character of zein gels (Figure 4 and Figure S3). In this case, the viscosity of the various formulations also showed a trend similar to that described for the samples prepared with fluorescein, but an increase in this parameter was evident when 5% of rhodamine B was added to the zein systems at 20% protein (Figure 5). This effect may be related to the different aqueous solubility of the molecule and to a certain interaction of the probe with the lipophilic residues of the protein. The described results confirm the significant influence exerted by a hydrophilic derivative on the elastic properties of zein gels.

**Figure 6:**
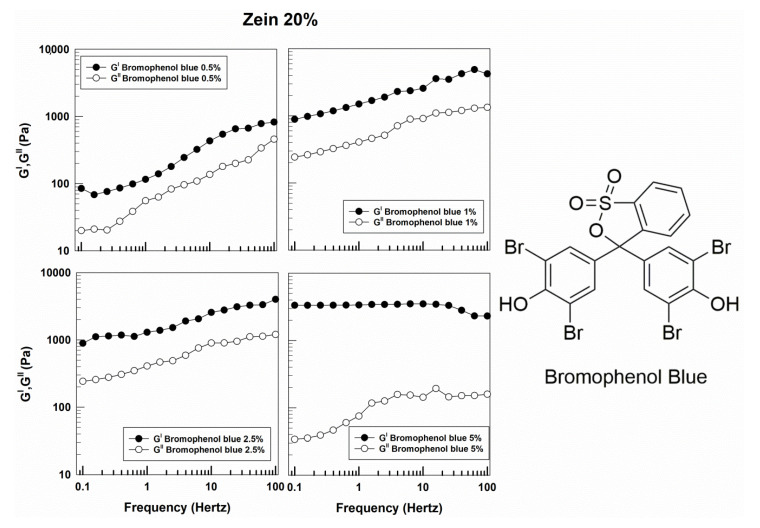
Evaluation of the elastic (G′) and viscous (G″) moduli of zein gels prepared using 20% *w*/*v* of protein and containing bromophenol blue (0.5%, 1%, 2.5%, 5% *w*/*w*) as a function of the frequency.

Conversely, the rheological features of zein gels containing a lipophilic compound such as bromophenol blue were quite different with respect to those of the formulations prepared with the hydrophilic molecules. Namely, increasing amounts of bromophenol blue added during the preparation of zein gels revealed a progressive gap between G′ and G″, but this trend is more noticeable at the highest concentration (5% *w*/*w*) of probe. In particular, both the storage and viscous moduli are completely independent of frequency, demonstrating a noticeable pseudo-plastic or shear-thinning behavior (Figure 6 and Figure S4). It was also interesting to note the effect of low concentrations of the lipophilic compound on the viscous and solid moduli that were characterized by a decreased gap with respect to the empty formulation and the system containing 5% of bromophenol blue, demonstrating that the rheological features of zein gels are dependent on the concentration of the lipophilic molecule. Indeed, the storage modulus of the 5% of lipophilic drug exceeds 1000 Pa, indicating that a greater amount of the compound promotes the formation of a stronger network and increases the stability of the gels [40].

**Figure 7:**
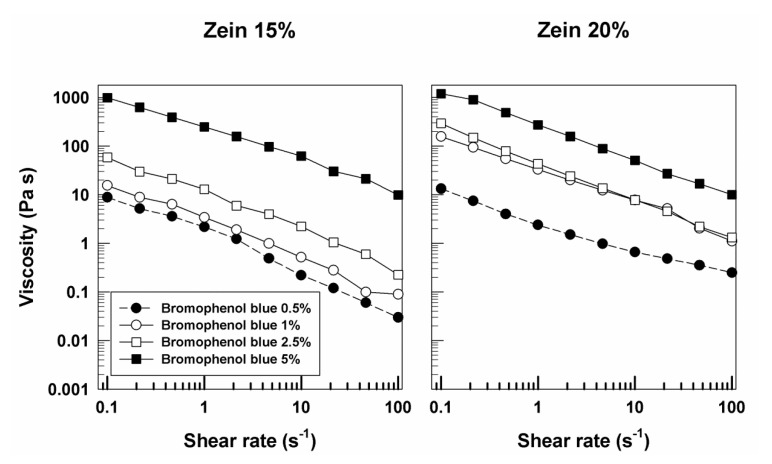
Evaluation of the viscosity of zein samples containing bromophenol blue as a function of the shear rate.

Moreover, as can be seen in Figure 7, the formulations containing bromophenol blue prepared with 20% of protein were characterized by a more noticeable solid-like character as compared to gels prepared with 15% of the biopolymer, showing a rapid decrease in viscosity when the shear rate increased (Figure 7). This was probably due to the peculiar chemical composition of the biopolymer, consisting of a predominance of lipophilic aminoacids, which could promote a better binding affinity with bromophenol blue as a consequence of the hydrophobic, electrostatic, and hydrogen interactions as proposed by Karthikeyan and coworkers [41].

**Figure 8:**
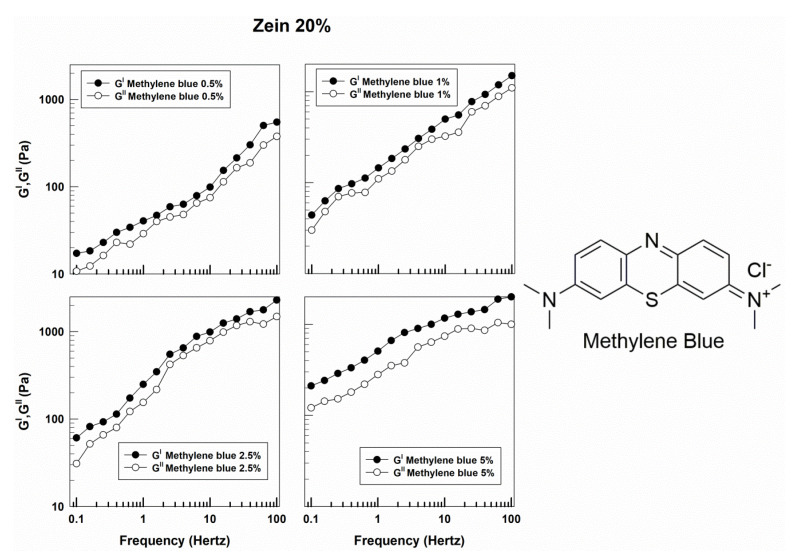
Evaluation of the elastic (G′) and viscous (G″) moduli of zein gels prepared using 20% *w*/*v* of protein and containing methylene blue (0.5%, 1%, 2.5%, 5% *w*/*w*) as a function of the frequency.

**Figure 9:**
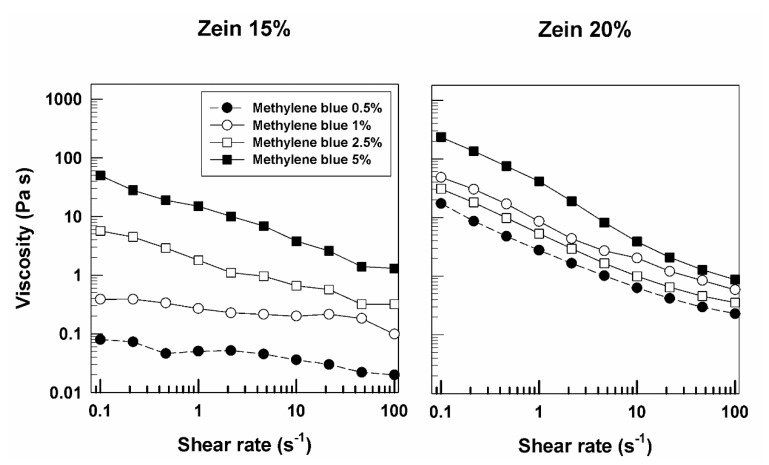
Evaluation of viscosity of zein samples containing methylene blue as a function of the shear rate.

As shown in Figure S5, the gels prepared with 15% *w*/*v* of zein containing low concentrations of methylene blue showed different rheological profiles when compared to the formulations described above; in fact, the viscous and the elastic moduli are close and often overlapped. Only at high values of frequency and concentrations of 2.5% and 5% of the compound does the solid character prevail (Figure S5). This behavior is more visible in the formulations prepared with a zein concentration of 20% *w*/*v*; a clear gap between the loss and storage moduli is obtained only when the highest amount of amphiphilic molecule was used (Figure 8). This is probably due to the different interactions that occur between the compound and the protein, suggesting that a high concentration of zein could promote a better retention of the molecule. This hypothesis was supported by the viscosity profiles of the formulations, prepared with 15% *w*/*v* of zein containing low concentrations of methylene blue, characterized by a relatively constant viscosity and a Newtonian behavior (Figure 9). Contrarily, the systems prepared with 20% *w*/*v* of protein, showed a progressive reduction in sample viscosity when the shear rate increased, demonstrating a pseudoplastic profile (Figure 9).

Moreover, the complex viscosity of the zein formulations containing the aforementioned model compounds was compared with the steady shear viscosity. As can be seen in Figures S6 and S7, the complex viscosity values of all the samples are very similar to those of the shear viscosity, confirming the rheological features of the zein systems that were previously described.

**Figure 10:**
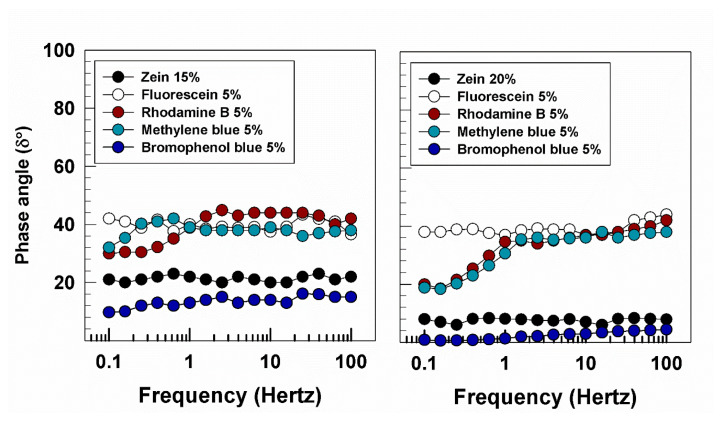
Phase angle of zein gels containing 5% of compounds characterized by different physico-chemical properties.

The modulation of the liquid/solid properties of zein gels as a function of the nature of the entrapped compound was summarized in Figure 10. Namely, the systems containing bromophenol blue showed the lowest phase angle, confirming their elastic properties, while the other formulations prepared with the hydrophilic compounds were characterized by a viscoelastic feature. The gels containing methylene blue showed a different profile as a function of the amount of zein used, ranging from a viscoelastic behavior when 15% of protein was used to strongly solid characteristics in the samples prepared with 20% of the biopolymer (Figure 10).

**Figure 11:**
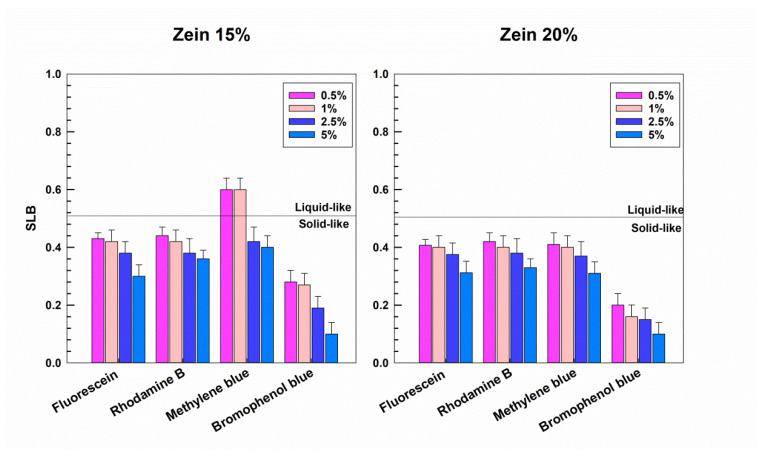
Values of the solid–liquid balance (SLB) of zein gels containing various compounds characterized by different physico-chemical properties as a function of the biopolymer concentration and the amount of probe used during the preparation of samples.

The SLB values of the various systems confirmed the behavior of the formulations that were previously described (Figure 11). In detail, the samples containing bromophenol blue showed low SLB values, indicating a clearly solid-like status of the formulations, while the systems containing methylene blue also evidenced, in this case, a different profile as a function of the amount of biopolymer and probe. In fact, the samples prepared with 15% of zein containing the lowest concentrations of methylene blue showed an SLB of ~0.6, and a slightly liquid character, while upon the increase in the protein concentration, a decrease in the SLB value occurred, demonstrating a more elastic behavior of the formulations. The zein samples containing the hydrophilic compounds (disodium fluorescein and rhodamine B) showed SLB values of less than 0.5 at both the protein concentrations, confirming their viscoelastic properties.

### 3.2. Evaluation of Release Profiles

**Figure 12:**
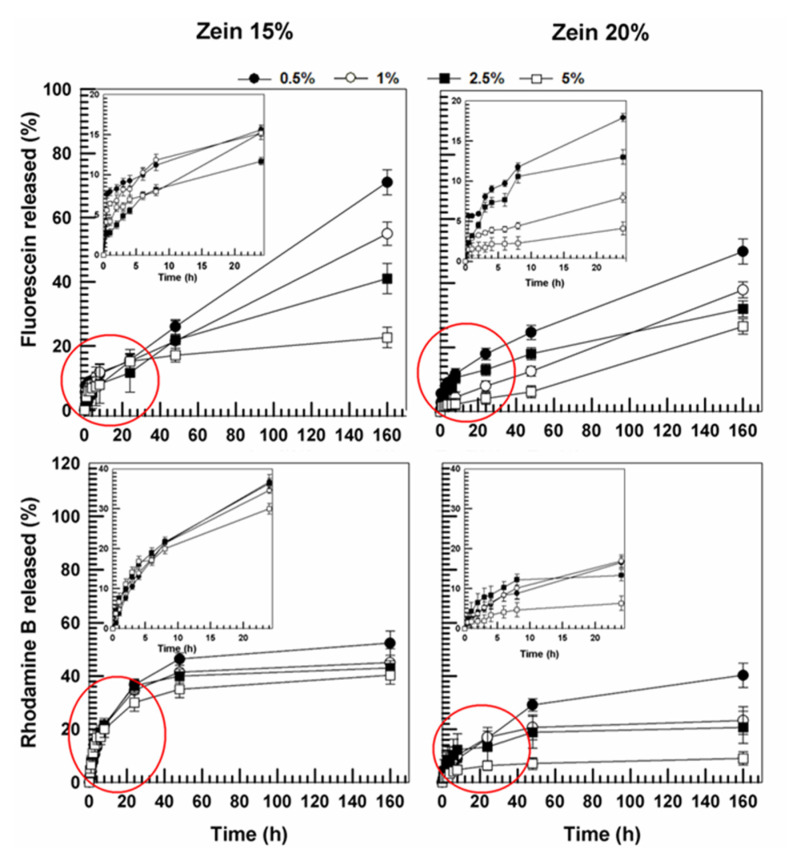
Release profile of disodium fluorescein and rhodamine B from zein gels (15%, 20% *w*/*v*) as a function of the amount of entrapped drug and time. Values represent the mean of three different experiments ± standard deviation.

The leakage of the model compounds from the zein formulations was investigated as a function of the biomaterial concentration and the incubation time in order to confirm the aforementioned rheological features of the systems. As shown in Figure 12, the leakage of disodium fluorescein and rhodamine B from zein-based gels in a PBS solution was constant and gradual over time, suggesting that the higher concentration of protein (20% *w*/*w*) can promote a better retention of the hydrophilic compounds. This is mainly due to the presence of polar residues on the zein network, which were able to promote the intramolecular hydrogen bonds with the hydroxyl- and amine-groups of the fluorescent probes [42]. It was interesting to note that the leakage of the molecules was inversely proportional to both the concentration of the probe and the quantity of protein used, demonstrating a strong interaction between the compound and the polymeric matrix.

**Figure 13:**
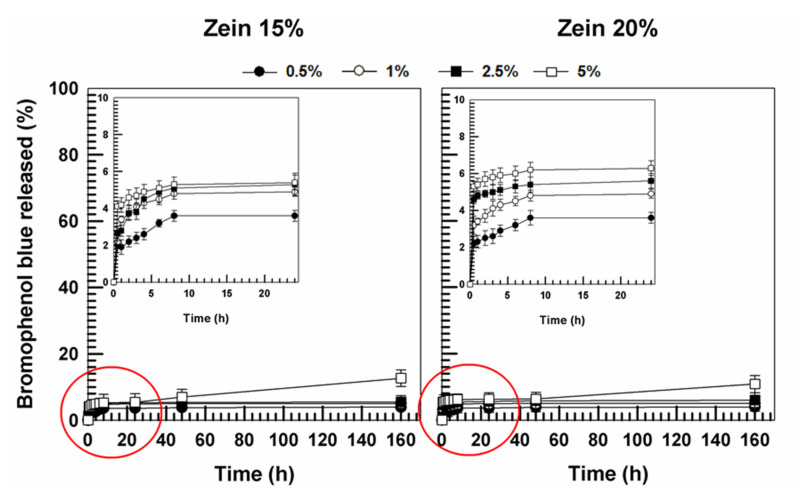
Release profile of bromophenol blue from zein gels (15%, 20% *w*/*v*) as a function of the amount of entrapped drug and time. Values represent the mean of three different experiments ± standard deviation.

On the contrary, the release profile of bromophenol blue from the protein matrix was not affected by the concentration of the protein. In detail, the probe leakage was constant and prolonged over time and was modulated by the amount of the entrapped compound (Figure 13). This trend can be related to the greater lipophilic character of the molecule, which is effectively retained in the polymeric structure by numerous hydrophobic interactions between the dibromophenyl residue and the apolar groups of zein, but also to the hydrogen bonds between the hydroxyl groups of the probe and the carbonyl residues of the protein. The slow leakage of bromophenol blue demonstrates a significant interaction between the probe and the protein-based gels.

**Figure 14:**
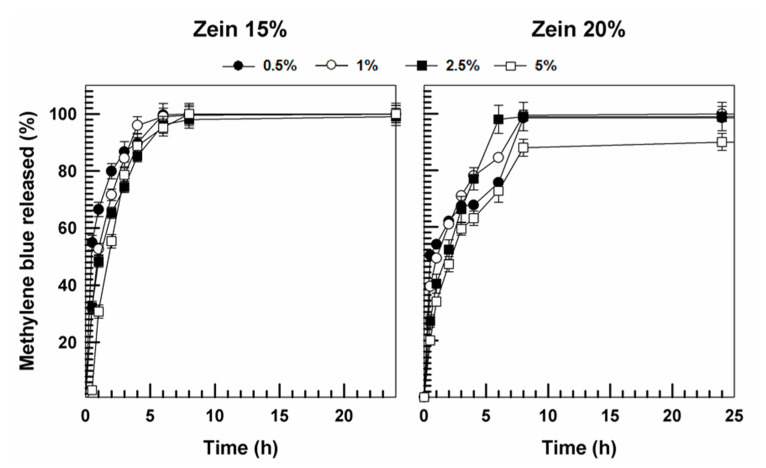
Release profile of methylene blue from zein gels (15%, 20% *w*/*v*) as a function of the amount of entrapped drug and time. Values represent the mean of three different experiments ± standard deviation.

The evaluation of the methylene blue release from the zein-matrix showed a different profile with respect to those previously described. Indeed, as shown in Figure 14, a great amount of the active compound was released from the zein formulations after a few hours. These findings are in agreement with those reported by Karthikeyan and coworkers, who showed by docking analyses that promethazine, an active compound characterized by a chemical structure similar to that of methylene blue, encapsulated in zein microspheres, is quickly released after only 2 h [41]. This is due to the preponderance of the hydrophobic interactions between the bioactive and specific aminoacid residues of protein and to the lack of hydrogen bonds, which prevents the effective retention of the molecule in the polymer network.

## 4. Conclusions

The scientific community is always looking for biopolymers from natural and sustainable sources in order to develop formulations which are able to encapsulate, protect, and release various active compounds in a controlled manner, modulating their pharmacological effects. In this scenario, zein demonstrated that it possesses peculiar structural properties useful for forming gels able to retain various biomolecules.

In this work, the influence of different model compounds on the rheological characteristics of zein-based gels was discussed. The addition of water-soluble compounds (rhodamine B and disodium fluorescein) and of a lipophilic probe (bromophenol blue) promoted a viscoelastic and solid-like behavior of the protein matrix, respectively. Zein gels showed a prolonged release of the probes, demonstrating a strong interaction between the entrapped compounds and the polymeric network. On the contrary, the systems prepared with methylene blue, an amphiphilic compound, were characterized by a different rheological profile influenced by the zein concentration and by a rapid release of the molecule after a few hours, suggesting a lesser ability of the gel network to retain the compound. The results demonstrate that the nature of the molecule to be entrapped in zein networks plays an essential role in determining the technological parameters of the polymeric structure. Zein formulations prepared using 15% and 20% *w*/*v* of protein can be potentially used as films, scaffolds and coating materials for various biomedical, food and pharmaceutical applications. For example, the rheological behavior of the proposed systems, characterized by a good degree of spreadability due to the low flow resistance when a shear rate is applied, can promote their use as biocompatible topical gels. The choice of suitable compounds (anti-inflammatory, antioxidant, antitumor, antiviral, etc.) to be retained as a function of the required outcome is mandatory in order to duly exploit the great versatility of this protein, and promoting the development of various low-cost formulations.

## Figures and Tables

**Table 1:** Chemical properties of the model compounds used.

Sample	Molecular Weight (g/mol)	Solubility in Water (g/L)	Log Pow	Source
Disodium fluorescein	376.27	500	−0.67	Merck
Rhodamine B	479.02	15	1.95	Merck
Methylene blue	319.86	25	5.85	LabChem
Bromophenol blue	669.96	Slightly soluble	9.2	Merck

## Supplementary Materials

The following are available online, Figure S1: Correlation between the strain (γ) and the elastic modulus (G′) of zein gels containing 5% of compounds characterized by different physico-chemical properties, Figure S2: Evaluation of the elastic (G′) and viscous (G″) moduli of zein gels prepared using 15% *w*/*v* of protein and containing sodium fluorescein (0.5%, 1%, 2.5%, 5% *w*/*w*) as a function of the frequency, Figure S3: Evaluation of the elastic (G′) and viscous (G″) moduli of zein gels prepared using 15% *w*/*v* of protein and containing rhodamine B (0.5%, 1%, 2.5%, 5% *w*/*w*) as a function of the frequency. Figure S4: Evaluation of the elastic (G′) and viscous (G″) moduli of zein gels prepared using 15% *w*/*v* of protein and containing bromophenol blue (0.5%, 1%, 2.5%, 5% *w*/*w*) as a function of the frequency. Figure S5: Evaluation of the elastic (G′) and viscous (G″) moduli of zein gels prepared using 15% *w*/*v* of protein and containing methylene blue (0.5%, 1%, 2.5%, 5% *w*/*w*) as a function of the frequency. Figure S6: Evaluation of complex viscosity (η*) of zein samples containing disodium fluorescein (A, B) and rhodamine B (C, D) as a function of frequency, Figure S7. Evaluation of complex viscosity (η*) of zein samples containing methylene blue (A, B) and bromophenol blue (C, D) as a function of frequency.
