# Supplementary material for: Influence of Various Model Compounds on the Rheological Properties of Zein-Based Gels
Source: Molecules. 2020 Jul 11;25(14):3174. doi: 10.3390/molecules25143174 (PMC7397198; doi:10.3390/molecules25143174)
Supplement: Supplementary file 1 [file molecules-25-03174-s001.pdf]

molecules

Influence of various model compounds on the rheological properties of zein-based gels

Agnese Gagliardi [1], Silvia Voci [2], Donatella Paolino[1], Massimo Fresta[2], Donato Cosco[2,*]

[1] Department of Experimental and Clinical Medicine, University "Magna Græcia" of Catanzaro, Campus Universitario "S. Venuta", I-88100, Catanzaro, Italy

[2] Department of Health Sciences, University "Magna Græcia" of Catanzaro, Campus Universitario "S. Venuta", I-88100, Catanzaro, Italy

* Correspondence: donatocosco@unicz.it; Tel.: +39-0961-369-4119

Received: date; Accepted: date; Published: date

Abstract: The controlled release of a compound entrapped in a biocompatible formulation is a sought-after goal in modern pharmaceutical technology. Zein is a hydrophobic protein which has several advantageous properties that make it suitable for use as a biocompatible and degradable material under physiological conditions. It is, therefore, being proposed for different biomedical and pharmaceutical applications. In particular, due to its gelling properties, it can be used to form a polymeric network able to preserve biomolecules from harsh environments. The current study was designed to investigate the influence of different probes on the rheological properties of gels made up of zein, in order to characterize the systems as a function of the polymer concentration. Four model compounds characterized by different physico-chemical properties were entrapped in zein gels, and different behavior (viscoelastic or pronounced solid-like characteristics) of the systems was observed. Zein-based gels showed various release profiles of the encapsulated compounds, suggesting that there are different interaction rates between the probes and the polymeric matrix.

Keywords: controlled release; gels; polymeric matrix; probes; rheology; zein.

Supplementary Materials

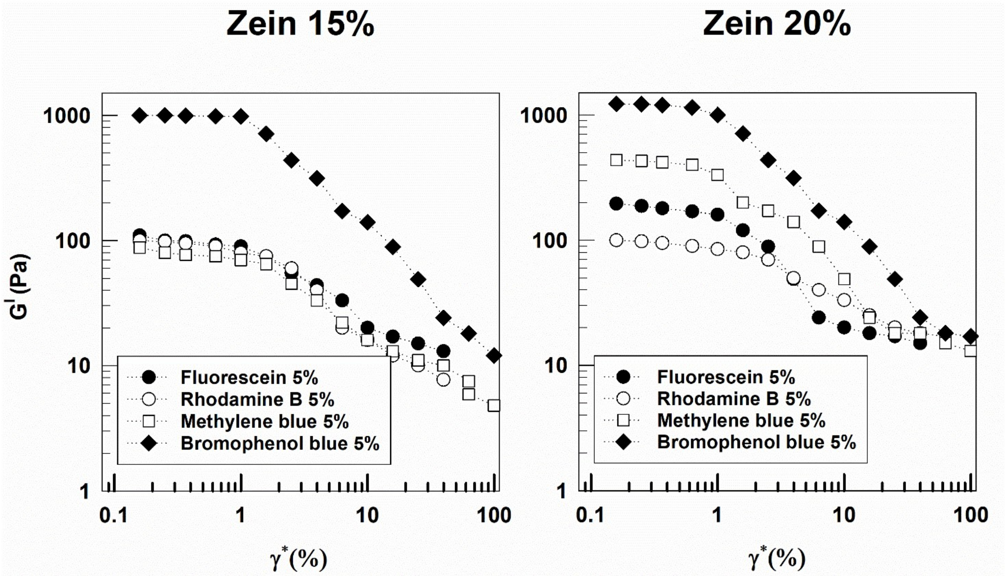

Figure S1. Correlation between the strain (γ) and the elastic modulus (G') of zein gels containing 5% of compounds characterized by different physico-chemical properties.

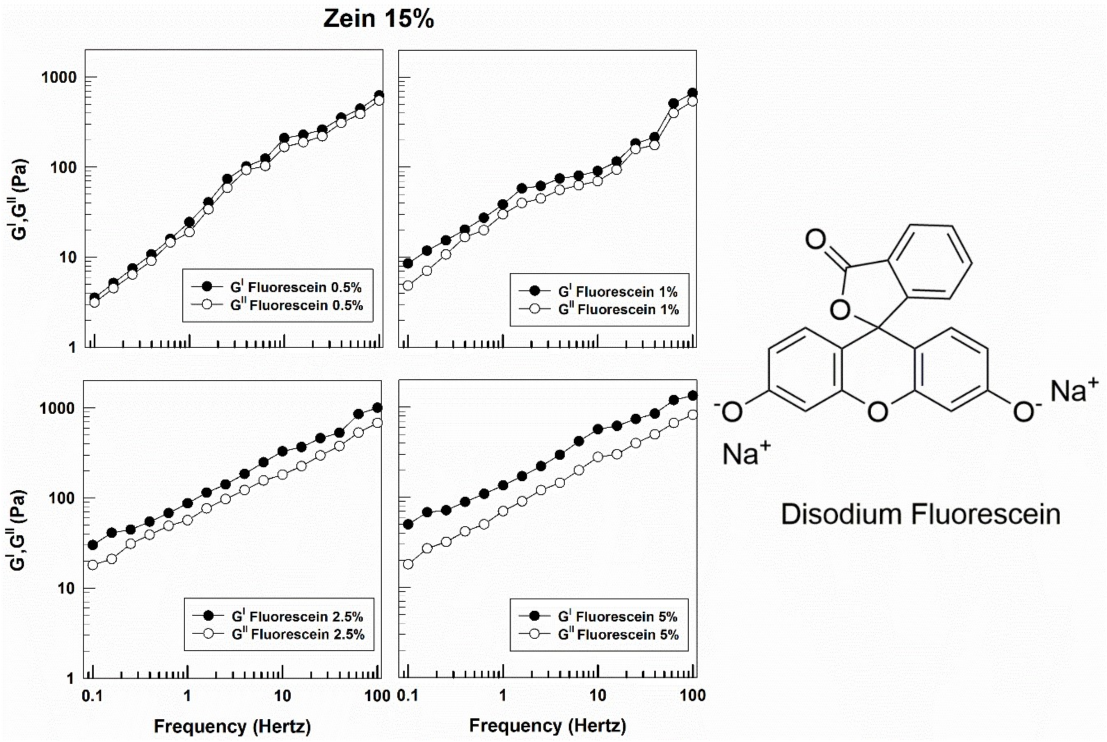

Figure S2. Evaluation of the elastic (G′) and viscous (G″) moduli of zein gels prepared using 15% w/v of protein and containing sodium fluorescein (0.5%, 1%, 2.5%, 5% *w* /*w*) as a function of the frequency.

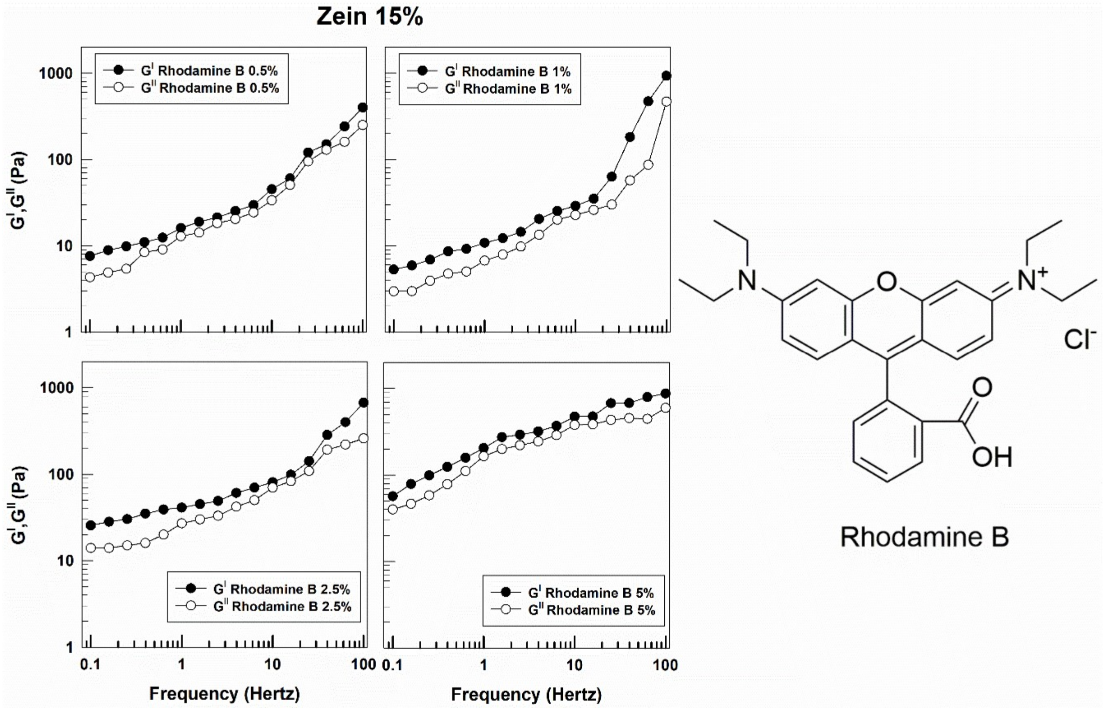

Figure S3. Evaluation of the elastic (G′) and viscous (G″) moduli of zein gels prepared using 15% *w*/*v* of protein and containing rhodamine B (0.5%, 1%, 2.5%, 5% *w* /*w*) as a function of the frequency.

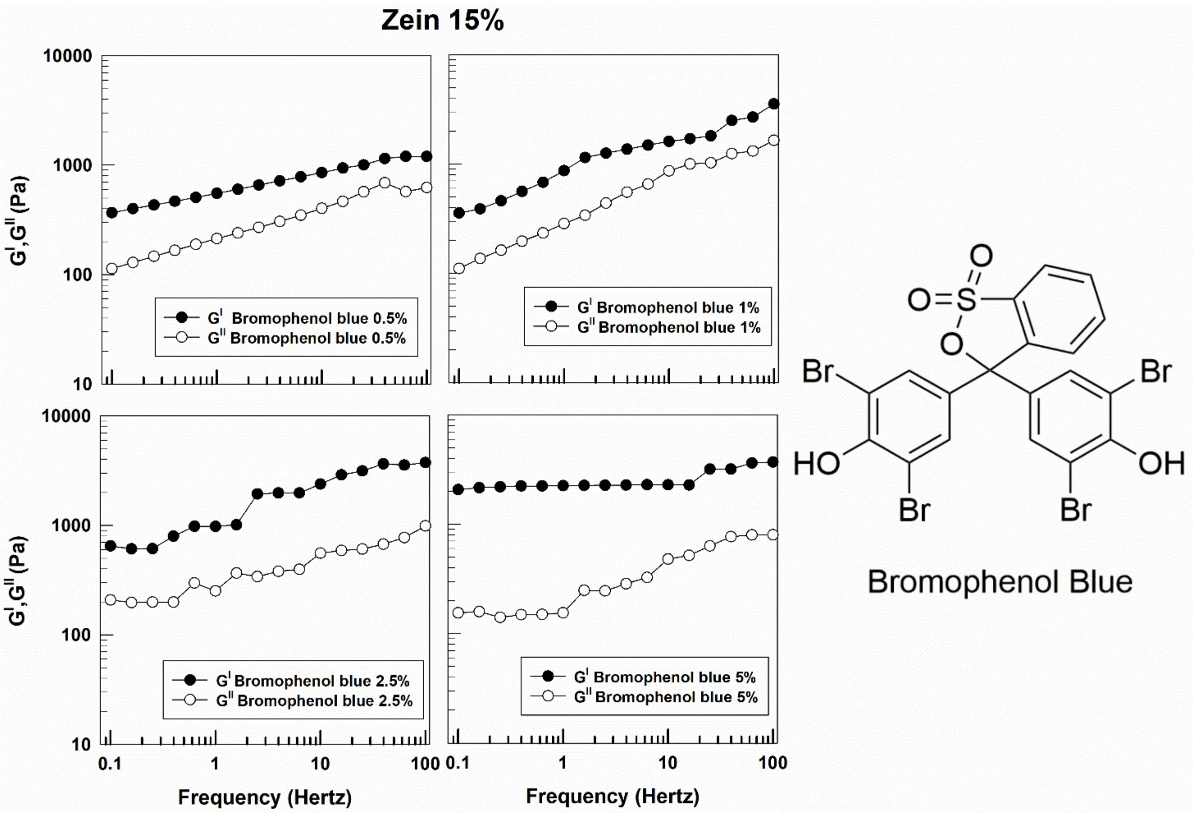

Figure S4. Evaluation of the elastic (G′) and viscous (G″) moduli of zein gels prepared using 15% *w*/*v* of protein and containing bromophenol blue (0.5%, 1%, 2.5%, 5% *w*/*w*) as a function of the frequency.

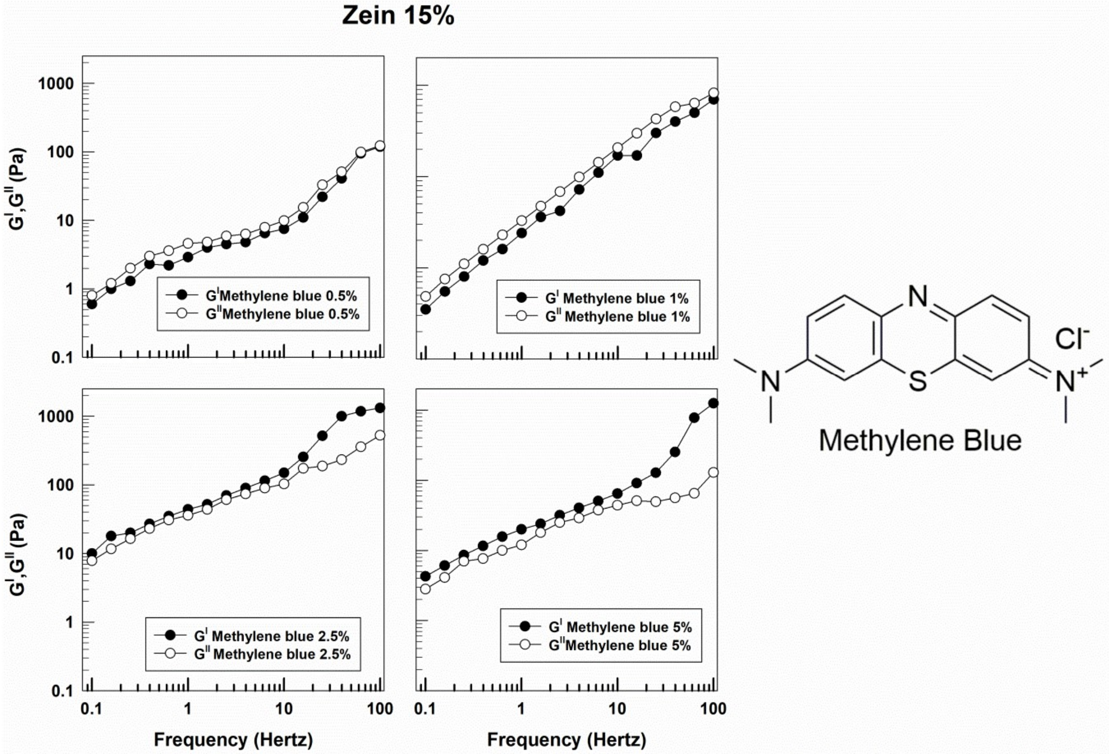

Figure S5. Evaluation of the elastic (G′) and viscous (G″) moduli of zein gels prepared using 15% *w*/*v* of protein and containing methylene blue (0.5%, 1%, 2.5%, 5% *w*/*w*) as a function of the frequency.

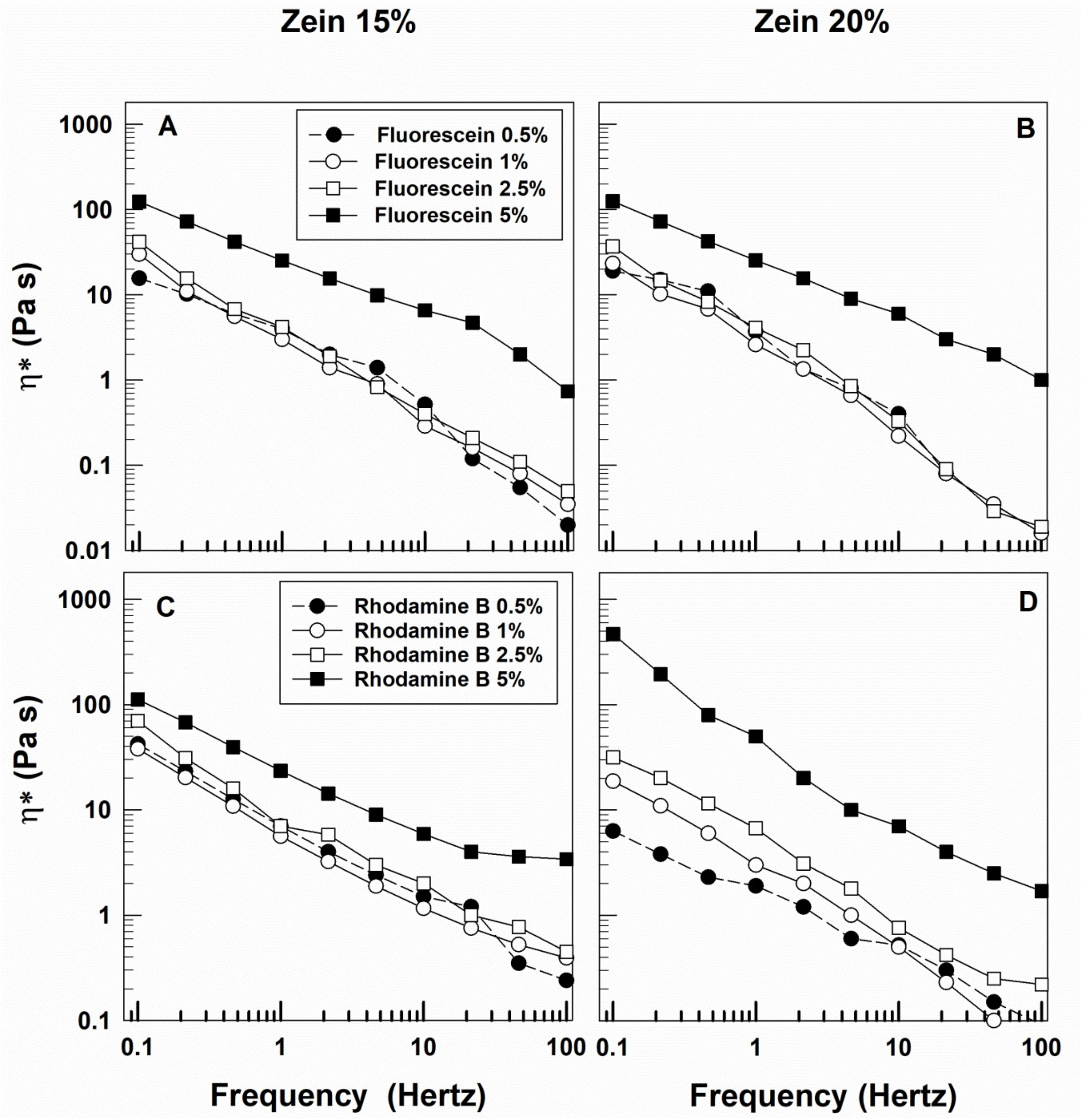

Figure S6. Evaluation of the complex viscosity (η*) of zein samples containing disodium fluorescein (**A**, **B**) and rhodamine B (**C**, **D**) as a function of frequency.

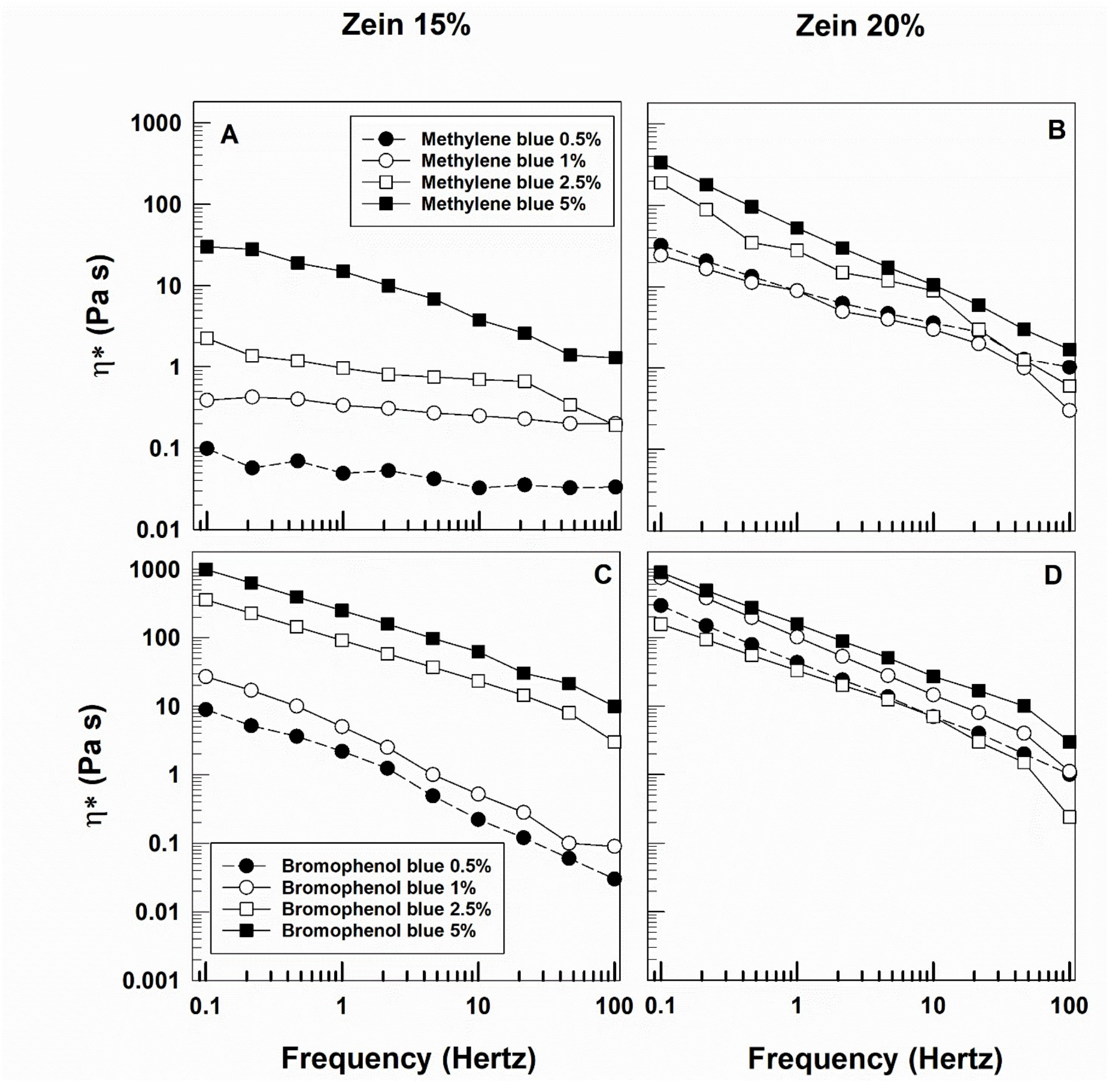

Figure S7. Evaluation of the complex viscosity (η*) of zein samples containing methylene blue (**A**, **B**) and bromophenol blue (**C**, **D**) as a function of frequency.

© 2020 by the authors. Licensee MDPI, Basel, Switzerland. This article is an open access article distributed under the terms and conditions of the Creative Commons Attribution (CC BY) license (http://creativecommons.org/licenses/by/4.0/).
